# Next-Generation Sequencing Combined With Conventional Sanger Sequencing Reveals High Molecular Diversity in *Actinidia* Virus 1 Populations From Kiwifruit Grown in China

**DOI:** 10.3389/fmicb.2020.602039

**Published:** 2020-12-16

**Authors:** Shaohua Wen, Guoping Wang, Zuokun Yang, Yanxiang Wang, Min Rao, Qian Lu, Ni Hong

**Affiliations:** ^1^Key Lab of Plant Pathology of Hubei Province, College of Plant Science and Technology, Huazhong Agricultural University, Wuhan, China; ^2^Key Laboratory of Horticultural Crop (Fruit Trees) Biology and Germplasm Creation of the Ministry of Agriculture, Wuhan, China

**Keywords:** next-generation sequencing, *Actinidia* spp., *Actinidia* virus 1, molecular diversity, recombination, RT-PCR

## Abstract

Kiwifruit (*Actinidia* spp.) is native to China. Viral disease–like symptoms are common on kiwifruit plants. In this study, six libraries prepared from total RNA of leaf samples from 69 kiwifruit plants were subjected to next-generation sequencing (NGS). *Actinidia* virus 1 (AcV-1), a tentative species in the family Closteroviridae, was discovered in the six libraries. Two full-length and two near-full genome sequences of AcV-1 variants were determined by Sanger sequencing. The genome structure of these Chinese AcV-1 variants was identical to that of isolate K75 and consisted of 12 open reading frames (ORFs). Analyses of these sequences together with the NGS-derived contig sequences revealed high molecular diversity in AcV-1 populations, with the highest sequence variation occurring at ORF1a, ORF2, and ORF3, and the available variants clustered into three phylogenetic clades. For the first time, our study revealed different domain compositions in the viral ORF1a and molecular recombination events among AcV-1 variants. Specific reverse transcriptase–polymerase chain reaction assays disclosed the presence of AcV-1 in plants of four kiwifruit species and unknown *Actinidia* spp. in seven provinces and one city.

## Introduction

The family Closteroviridae contains many plant viruses causing different diseases of economically important crops (Martelli et al., 2012). Viruses in the family Closteroviridae are characterized by having 650–2,200 nm, flexuous filamentous virions consisting of a positive-sense, single-stranded (+ss) RNA with size ranging from 13 to 19.3 kb (Agranovsky et al., 1995; Rubio et al., 2013). According to the International Committee on Taxonomy of Viruses (ICTV) report posted on August 2018 (ICTV Master Species List, 2018 v1), the family Closteroviridae comprises more than 50 recognized species, which are classified into four genera, *Closterovirus*, *Ampelovirus*, *Crinivirus*, and *Velarivirus*, and seven unassigned members, based on their vectors and phylogenetic relationships (Karasev, 2000; Dolja et al., 2006). Members of the family Closteroviridae share a common genome organization, containing a replication-related module encoded by open reading frame (ORF) 1a and 1b, and a five-gene module encoding a small hydrophobic protein, a homolog of the plant heat shock protein HSP70 (HSP70h), a ∼60 kDa protein, a major coat protein (CP), and a minor coat protein (CPm) (Martelli et al., 2012). A notable exception is that viruses in the genus *Ampelovirus* have smaller genomes, which do not possess CPm and have unique ORFs. Previous studies showed that viruses in the family Closteroviridae have great genetic variability, and molecular evolution might be necessary for adaptation to new environments (Holland et al., 1982; Kreuze et al., 2002; Cuellar et al., 2008; Bertazzon et al., 2010; García-Arenal and Fraile, 2011; Esteves et al., 2012). Homologous recombination has been described for CP and P20 genes of citrus tristeza virus (CTV) (Rubio et al., 2001, 2013; Bar-Joseph and Mawassi, 2013), a member in the genus *Closterovirus*, for CP genes of ampeloviruses grapevine leafroll-associated virus 3 (GLRaV-3) (Turturo et al., 2005; Farooq et al., 2012), GLRaV-1 (Fan et al., 2015), and GLRaV-11 (Boulila, 2010), for HSP70h gene of plum bark necrosis stem pitting-associated virus (PBNSPaV) (Qu et al., 2014), and for ORF1a of GLRaV-4 (Adiputra et al., 2019).

Kiwifruit (*Actinidia* spp.) is an economically important fruit crop cultivated worldwide. Since the first report of apple stem grooving virus (ASGV) (Clover et al., 2003) naturally infecting kiwifruit, currently at least 19 viruses infecting kiwifruit have been reported (Pearson et al., 2011; Chavan et al., 2012, 2013; Blouin et al., 2013, 2018; Wang et al., 2016, 2020; Zheng et al., 2017; Veerakone et al., 2018; Wang D. et al., 2018; Wang Y. et al., 2018; Zhao et al., 2019, 2020). Among these viruses, *Actinidia* virus 1 (AcV-1), a tentative member of the family Closteroviridae, was first characterized from *Actinidia chinensis* in New Zealand (Blouin et al., 2018). The reported genome of AcV-1 isolate K75 (GenBank accession no. KX857665) consists of an 18,848 nt + ss RNA containing at least 12 ORFs. ORF1a encodes a multifunctional protein with two papain-like leader protease (L-Pro) domains (L1 and L2), one methyltransferase (Mtr) domain, and one helicase (Hel) domain. ORF 1b, expressed by a + 1 ribosomal frameshift, encodes an RNA-dependent RNA polymerase (RdRp). ORF2 and ORF3 encode two hypothetical proteins with unknown functions and predicted molecular masses of 13.6 and 25.4 kDa, respectively. ORFs 4–8 consist of a five-gene module, in which, instead of CPm in the module of most viruses in the family Closteroviridae, ORF7 encodes a 30 kDa protein (p30) with a conserved domain of thaumatin-like proteins. ORFs 9–11 encode three small proteins, whose functions are currently unknown. The virus was reported in China in 2018, the second country with AcV-1 infecting kiwifruit (Peng et al., 2018; Wen et al., 2018).

Until now, only one complete genome sequence of AcV-1 isolate K75 has been documented (Blouin et al., 2018), and a few sequences of the viral CP and HSP70h genes are available in GenBank. These sequences are valuable for designing molecular diagnostic methods. The relationship between the infection with the virus and occurrence of kiwifruit disease is still unknown, and the viral populations remain poorly understood. In this study, for the first time, the genome-wide genetic diversity of AcV-1 variants from kiwifruit plants grown in China was determined by using next-generation sequencing (NGS) combined with conventional Sanger sequencing for reverse transcriptase–polymerase chain reaction (RT-PCR) products. This study provides useful information for understanding the molecular evolution within AcV-1 populations and developing reliable molecular detection methods of the virus, which is necessary for epidemiological investigation of the viral disease.

## Materials and Methods

### Plant Materials

During the growing season of kiwifruit plants in 2016–2019, leaf samples of 245 kiwifruit plants, including 104 plants of *A. chinensis*, 39 *Actinidia delicious* plants, nine *Actinidia arguta* plants, 36 *Actinidia eriantha* plants, one *Actinidia rufa* plant, and 56 plants of unknown species, were collected in Hubei, Yunnan, Jiangxi, Shandong, Zhejiang, Henan, Fujian, and Shanxi provinces and Chongqing and Shanghai cities, covering the major kiwifruit production areas in China. Of these plants, 231 plants showed virus disease–like symptoms, including uneven chlorosis between veins, mosaic, ringspot, chlorotic spot, and/or deformation, whereas 14 plants were asymptomatic. Leaves from each plant were pooled as one sample named with geographic origin followed by a plant number. The presence of six known viruses AcV-1, *Actinidia* virus A (AcVA), *Actinidia* virus B (AcVB), ASGV, *Actinidia* chlorotic ringspot-associated emaravirus (AcCRaV), and citrus leaf blotch virus (CLBV), in these samples were tested by RT-PCR using specific primers reported previously (James, 1999; Harper et al., 2008; Blouin et al., 2012; Zheng et al., 2017; Supplementary Table S1). A leaf sample of a virus-free seedling of *A. chinensis* was used as a negative control.

According to the origins and symptoms of individually collected leaf samples as mentioned above, six samples were prepared for RNA-seq analyses (Supplementary Table S2). One leaf sample (ID: Z2) from an *A. delicious* plant showing severe chlorotic spots, leaf mottle, and deformation, grown at a field in Henan province, and one sample (ID: JX5) from an *A. chinensis* plant showing leaf yellowing from Jiangxi province were individually used for RNA-seq analyses. Additionally, in order to have a full view of kiwifruit virome, four pooled samples JS, ZZ, CQ, and JX were also subjected to RNA-seq analyses. The sample JS was a mixture of four leaf samples (JS27, JS29, JS30, and JS45) from Hubei province. The sample ZZ consisted of leaf samples from 30 kiwifruit plants (ZZ1–ZZ11, ZZ13–ZZ31) grown at a field in Henan province. The sample CQ consisted of leaf samples from 28 kiwifruit plants (CQ1–CQ28) grown at a field in Chongqing city. The sample JX consisted of leaf samples from five kiwifruit plants (JX1–JX4 and JX6) grown at a field in Jiangxi province. Most of these RNA-seq–analyzed samples (except for two asymptomatic samples JX6 and CQ18) showed viral disease–like symptoms as listed in Supplementary Table S4.

Additionally, one leaf sample (ID: WH4) from an *A. arguta* plant showing severe leaf mottle and deformation, grown at a field in Hubei province, was used for Sanger sequencing of AcV-1 genome.

### RNA-Seq

Total RNAs were extracted from the six samples prepared for RNA-seq analyses as mentioned above, and ribosomal RNAs (rRNAs) were removed using an Epicenter Ribo-Zero rRNA removal kit (Epicenter, Madison, WI, United States). The rRNA-depleted RNA extracts were used for cDNA library construction with a TruSeq RNA Sample Prep Kit v2 (Illumina, San Diego, CA, United States) and sequenced on an Illumina HiSeq XTen sequencing machine with a paired-end 150 bp setup (Biomarker Biology Technology Ltd., Company, Beijing, China).

Raw reads were trimmed of adaptor sequences and filtered for low-quality reads with more than 5% ambiguous bases (Ns) or with more than 10% of the bases below Q20 quality as described previously (Liu et al., 2020). Qualified reads were *de novo* assembled into contigs using the CLC Genomics Workbench 11.0 (Qiagen, Valencia, CA, United States) platform. Contigs were subsequently screened for sequence identity against the NCBI databases^1^ using BlastX and BlastN programs.

### Determination of Complete Genomic Sequences of AcV-1 Isolates

The complete genomic sequence of one AcV-1 variant (JS27) from sample JS27 and the near-full-length genomic sequence of one AcV-1 variant (Z2) from sample Z2 were individually determined by RT-PCR using primers (Supplementary Table S3) designed on the basis of the contig sequences derived from the RNA-seq and/or the sequence of the reported isolate K75. The complete genomic sequence of AcV-1 variant WH4-2 and the near-full-length genomic sequence of AcV-1 variant WH4-1 from sample WH4 were individually determined by RT-PCR using primers (Supplementary Table S3) designed on the basis of available AcV-1 sequences. Most of the adjacent fragments overlapped by more than 100 bp. The 5′ and 3′ terminal sequences of the viral genomic RNA were determined by a RACE strategy using a commercial kit (Invitrogen GeneRacer Kit, United States) according to the manufacturer’s instructions and specific reverse primers (Supplementary Table S3). RT-PCR solutions and conditions were like those reported previously (Zheng et al., 2017), except that annealing temperature and extension time varied, depending on the primer sets used in each reaction and the sizes of PCR products. Sequences of RT-PCR fragments from each sample were assembled into a continuous sequence using DNAMAN6.0 (Lynnon, Quebec, Canada).

### RT-PCR Detection of AcV-1

The primer set cp-F/cp-R (5′-TGAGCTRGGRATAGATGTTGC-3′/5′-TCTCTCAGGGTTMGGATGAGT-3′) (Wen et al., 2018) designed on the basis of the conserved sequence of the CP gene (positions 16,607–16,627 nt/16,962–16,982 nt of isolate K75 genome) of AcV-1 was used for the specific detection of AcV-1 in kiwifruit leaf samples. Total RNA was extracted from the collected leaf materials (each about 0.5 g) using a CTAB method (Fan et al., 2015). The RNA was diluted in 40 μL RNase-free water. The 20-μL cDNA synthesis solution contained 9 μL RNA template, 1 μL p(N)6 random primer, 0.5 μL Moloney murine leukemia virus (M-MLV) reverse transcriptase, 0.5 μL recombinant RNase inhibitor (Takara, Dalian, China), 4 μL 5 × M-MLV buffer, 1 μL 2.5 mM dNTP, and 4 μL RNase-free water. A 25 μL PCR reaction solution contained 2.0 μL of the cDNA template, 2.5 μL of 10 × reaction buffer, 1.5 μL of 2.5 mM dNTP, 0.5 μL each of 10 mM primers, 0.25 μL r*Taq* (5 U/μl) (TakaRa, Dalian, China), and 17.75 μL ddH_2_O. The cycling parameters were set as follows: denaturation at 95°C for 5 min, followed by 35 cycles of denaturation (30 s at 95°C), annealing (30 s at 54°C), and extension (45 s at 72°C). The PCR products were separated on 1.2% agarose gel, stained with ethidium bromide, and visualized under UV light.

### Cloning and Sequencing of RT-PCR Products

PCR products were gel purified, ligated into the pMD18-T vector (Takara, Dalian, China), and subsequently transformed into cells of *Escherichia coli* strain Top10. Positive clones were identified by PCR using the *E. coli* cultures as templates. At least three positive clones of each PCR product were sequenced at Shanghai Sangon Biological Engineering & Technology and Service Co., Ltd., Shanghai, China.

### Sequence Analyses

ORFs were predicted using the Open Reading Frame Finder at https://www.ncbi.nlm.nih.gov/orffinder/. Conserved domains in predicted proteins were identified by using the Conserved Domain Database (CDD)^2^ websites in NCBI. Multiple alignments and identity analyses of nucleotide and amino acid sequences were performed using the ClustalW2 program^3^. Phylogenetic trees were constructed using the neighbor-joining method with 1,000 bootstrap in MEGA 7.0.

### Recombination Analysis

Genomic sequences of AcV-1 variants were examined for potential recombination events using the Recombination Detection Program (RDP) version RDP4.94 with default parameter settings (Martin et al., 2015). The RDP software includes the RDP, GENECONV, BOOTSCAN, MaxChi, Chimaera, and SISCAN methods. AcV-1 genomic sequences were aligned in MEGA v.7.0.1 software and exported to the RDP program to generate evidence of recombination. At a Bonferroni-corrected *p*-value cutoff ≤ 0.05, recombinant sites detected by four or more algorithms in the RDP were considered as recombination events.

## Results

### Viruses Identified by RNA-Seq Analysis

In total, 67,640,546, 92,477,928, 73,387,306, 82,730,664, 79,782,996, and 73,419,336 clean reads were obtained and 177,848, 315,308, 165,312, 246,566, 221,290, and 174,235 unique contigs were generated from RNA-seq samples JS, ZZ, Z2, CQ, JX, and JX5, respectively (Supplementary Table S2). BlastX searches against the NCBI NR database revealed that 254, 154, 137, 620, 73, and 18 contigs (accounting for 0.01–0.25% of total contigs) from the six samples respectively matched with viral proteins (Supplementary Table S2). AcV-1 was recovered from all these RNA-seq samples, and 121 contigs were assigned to this virus, accounting for 9.63% of the total viral contigs (Table 1). Meanwhile, five other well-documented kiwifruit viruses AcCRaV, AcVA, AcVB, CLBV, and *Actinidia* seed-borne latent virus (ASbLV) (Veerakone et al., 2018) were identified in sample JS. Four other viruses, including ASGV, *Actinidia* emaravirus 2 (AcEV-2) (Wang et al., 2020), and two unreported kiwifruit-infecting viruses, belonging to families Betaflexiviridae and Secoviridae, were identified in samples ZZ and Z2. In sample CQ, eight viruses, including AcV-1, AcCRaV, ASbLV, AcVA, AcVB, CLBV, ASGV, and an unreported virus in the family Secoviridae, were identified. In sample JX, five known viruses, including AcV-1, ASbLV, AcVA, AcVB, and CLBV, were identified. In sample JX5, only one known virus AcV-1 was identified. Additionally, a virus potentially belonging to the family Bromoviridae was identified in samples JX and JX5. Here, only AcV-1 sequences were considered for further analyses.

**TABLE 1:** Viruses and their sequences identified from six kiwifruit samples by using RNA-seq analysis.

Virus^a^	JS		ZZ		Z2		CQ		JX		JX5	
						
	Reads	Contigs	Reads	Contigs	Reads	Contigs	Reads	Contigs	Reads	Contigs	Reads	Contigs
*Actinidia* virus 1	34,773	16	1,136	18	3,153	26	9,586	37	3,014	14	7,896	10
*Actinidia* chlorotic ringspot-associated emaravirus	27,982	34	11,820	5	72	5	75,516	33				
*Actinidia* seed-borne latent virus	46,066	12	12	2	27	2	70,643	28	24,626	2		
*Actinidia* virus A	9,149	13	3,347	32	11,986	13	104,048	57	966	12		
*Actinidia* virus B	38,338	16	706	9	18,297	4	317,494	27	55	4		
Apple stem grooving virus			4,947	11	19	5	48,357	20				
Citrus leaf blotch virus	107,584	32	102,543	3	72	8	54,632	3				
*Actinidia* emaravirus 2			1,034	15	28,572	4						
Bromoviridae									192,019	24	189	5
Betaflexiviridae			19	3	31	4						
Secoviridae			2,333	1	234	2	277,845	14				

^*a*^*Three virus species, which are firstly identified in kiwifruit samples, are indicated by their family names.*

### AcV-1 Sequences Identified by RNA-Seq Analysis

**FIGURE 1:**
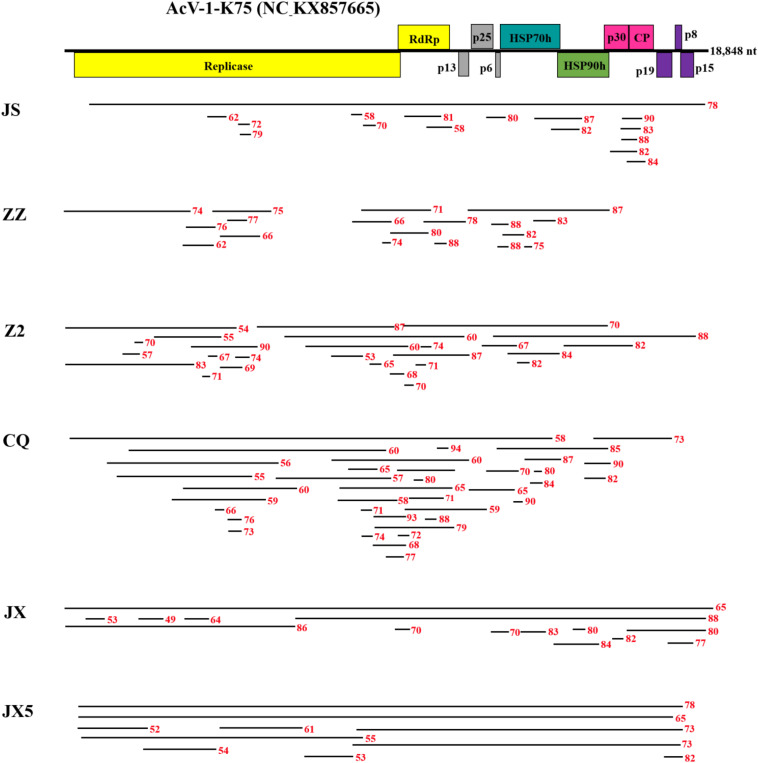
Contigs with significant nucleotide sequence identity to *Actinidia* virus 1 (AcV-1) isolate K75. A schematic diagram of the genome organization of AcV-1 isolate K75 was used as reference showing the contig positions. Six samples JS, ZZ, Z2, CQ, JX, and JX5 were named with the abbreviation of their geographic origins. Black lines represent AcV-1 contigs and their position at the genome of isolate K75. Red numbers indicate the nucleotide identity between each contig and the corresponding sequence of AcV-1 isolate K75.

From sample JS, 16 contigs matched the genomic sequence of AcV-1. One contig of 18,779 nt was near the full length of AcV-1 genome sequence, while the remaining contigs were 395–1,467 nt long. These contigs shared 58.0–90.0% nt sequence identity with corresponding sequences of isolate K75 (Figure 1). Comparison of these short contigs and the 18,779 nt contig revealed 59.6–92.9% nt sequence identity with each other.

From sample ZZ, 18 AcV-1 contigs with sizes ranging from 224 to 4,132 nt covered 71.5% of the viral genome and displayed 62.1–88.2% nt sequence identity with corresponding sequences of AcV-1 isolate K75 (Figure 1).

From sample Z2, 26 contigs ranging from 248 to 6,041 nt in length matched the genomic sequence of AcV-1, with a total coverage of 97.9%. These contig sequences shared 53.1–88.2% nt identity with corresponding sequences of isolate K75 (Figure 1).

From sample CQ, 37 contigs matched the genomic sequence of AcV-1. One contig (named CQ-c13707) was 13,707 nt in length and covered the ORF1a-ORF4 (position at 248–14,118 nt) of the viral genomic sequence. The remaining contigs were 212–7,590 nt in length. These contigs shared 55.2–94.3% nt sequence identity with corresponding sequences of isolate K75 (Figure 1).

From sample JX, 14 contigs matched the genomic sequence of AcV-1. One large contig (named JX-c18869) had a size of 18,869 nt, nearly covering the full-length of AcV-1 genome sequence, and another contig (named JX-c12007) was 12,007 nt (position at 6,772–18,873 nt) covering about 64% of the viral genomic sequence. The remaining contigs were 311–6,776 nt in length. All these contigs shared 49.0–88.3% nt sequence identity with corresponding sequences of isolate K75 (Figure 1).

From sample JX5, 10 contigs matched the genomic sequence of AcV-1. Two contigs (named JX5-c18818 and JX5-c18410) were 18,818 and 18,410 nt in length, respectively, and covered nearly the complete genome of AcV-1. Meanwhile, another two contigs (named JX5-c10087 and JX5-c10084) matched approximately in the same position (8,710–18,800 nt) of the viral genome. The four contigs shared 63.3–86.4% nt sequence identity with each other and 65.2–78.1% nt sequence identity with corresponding sequences of isolate K75. The remaining six contigs were 667–8,857 nt and shared 54.4–78.3% nt sequence identity with corresponding sequences of AcV-1 isolate K75 (Figure 1).

For the following analyses, each contig derived from RNA-seq analyses was named as a sample name followed by the contig length, and each sequence was considered as a molecular variant.

### Genomic Characteristics and Sequence Diversity of Chinese AcV-1 Variants

A complete genomic sequence (JS27, 18,896 nt, GenBank accession no. MT936297) of AcV-1 from the sample JS27 was reconstructed by Sanger sequencing of RT-PCR and 3′ and 5′ RACE products using primers designed on the basis of sequence of a large contig (18,779 nt) derived from RNA-seq. In addition, a near-full-length genomic sequence (Z2, 18,685 nt, excluding about 300 nt at the 3′ terminus) (GenBank accession no. MT936306) of AcV-1 infecting an *A. delicious* plant (ID: Z2) was reconstructed by RT-PCR using primers designed on the basis of sequences in the RNA-seq contigs. The two reconstructed genomic sequences showed greater than 98.4% identity at nt level with corresponding contig sequences generated from RNA assembly, indicating that the assembled contig sequences from RNA-seq data were reliable. Meanwhile, a complete genomic sequence of 18,851 nt (WH4-2) (GenBank accession no. MT936305) and a near-full-length genomic sequence (WH4-1, 16,871 nt, excluding about 2,000 nt at the 5′ terminus) (GenBank accession no. MT936304) of AcV-1 infecting an *A. arguta* plant (ID: WH4) were determined by Sanger sequencing of RT-PCR and 3′ and 5′ RACE products using primers designed on the basis of all available sequences.

These sequences together with near-full-length genomic sequences of three contigs (JX-c18869, JX5-c18818, and JX5-c18410) (GenBank accession nos. MT936303, MT936301, and MT936300), four contigs (CQ-c13707, JX-c12007, JX5-c10087, and JX5-c10084) (GenBank accession nos. MT936296, MT936302, MT936299, and MT936298) with sizes > 10,000 nt obtained from RNA-seq, and the genomic sequence of K75 were compared with each other (Table 2). The genome-wide nt sequence identity among the Chinese AcV-1 variants was about 60–87%. All these sequences showed 68.3–79.3% nt identity with that of isolate K75 reported from New Zealand. Moreover, high diversity was present in the AcV-1 sequences derived from the same sample. The two variants WH4-1 and WH4-2 from sample WH4 showed 62.6% nt sequence identity with each other, and the four contigs JX5-c18818, JX5-c18410, JX5-c10087, and JX5-c10084 from sample JX5 showed 63.3–86.5% nt sequence identity with each other. The genome structure of these Chinese AcV-1 variants was identical to that of K75 and consisted of 12 ORFs. There was variation in the length of the 5′ untranslated region (UTR) of genomic RNA among the Chinese AcV-1 variants and isolate K75. The 5′-UTR lengths of three AcV-1 variants Z2, JS27, and WH4-2 were 416, 290, and 288 nt, with 39.7, 42.9, and 78.3% nt sequence identities between Z2 and WH4-2, Z2 and JS27, and WH4-2 and JS27. These sequences shared 64.3–79.3% identity with 304-nt 5′-UTR of K75. The 3′-UTR lengths of AcV-1 variants JS27, WH4-2, and WH4-1 were 253, 252, and 252 nt, which were about 152 nt shorter than the 404-nt 3′-UTR of K75. The 3′-UTR of these Chinese AcV-1 variants shared 74.1–92.1% nt sequence identity with each other and 80.0–88.1% nt sequence identity with that of K75.

**TABLE 2:** Nucleotide and amino acid sequence comparison of the genome, ORFs, and UTRs of 11 *Actinidia* virus 1 (AcV-1) variants determined in this study with the corresponding sequences of isolate K75.

Vatiants	Genome		5′UTR		ORF1a			ORF1b (RdRp)			ORF2 (p13)			ORF3 (p25)			ORF4 (p6)			ORF5 (HSP70h)		
								
	nt	%	nt	%	nt	nt%	aa%	nt	nt%	aa%	nt	nt%	aa%	nt	nt%	aa%	nt	nt%	aa%	nt	nt%	aa%
**K75**	18,848	—	304	—	9,558			1,533			339			675			156			1,755		
Z2	18,685	68.3	416	64.3	9,597	61.6	61.1	1,527	72.2	80.9	366	56.6	48.2	675	58.4	51.3	156	76.9	86.3	1,755	81.0	88.0
**JS27**	18,896	79.3	290	77.2	9,600	72.1	74.2	1,527	83.3	92.9	339	85.6	74.1	675	86.4	85.3	156	89.1	94.1	1,755	88.0	92.1
**WH4-2**	18,851	79.2	288	79.3	9,561	73.2	75.0	1,527	82.2	91.9	339	79.7	74.1	675	85.6	85.7	168	89.1	90.2	1,755	88.7	93.0
WH4-1	16,871	70.6	—	—	—	—	—	1,527	70.4	79.7	366	54.6	48.2	675	58.4	51.8	156	82.1	86.3	1,755	86.1	91.3
CQ-c13707	13,707	63.6	—	—	9,666	60.3	59.4	1,527	71.3	79.9	366	52.5	41.1	768	56.2	55.8	156	82.1	88.2	—	—	—
JX-c18869	18,869	68.9	—	—	9,609	60.1	59.1	1,527	71.7	81.5	366	57.5	44.6	675	56.9	50.5	156	82.7	86.3	1,755	84.1	90.2
JX-c12007	12,007	87.3	—	—	—	—	—	1,527	90.4	94.5	339	80.8	72.3	675	84.6	84.8	156	89.1	92.2	1,755	88.4	93.5
JX5-c18818	18,818	79.2	—	—	9,534	72.7	74.9	1,527	81.3	93.5	339	79.4	71.4	675	83.4	81.7	168	90.4	94.1	1,755	88.2	94.2
JX5-c18410	18,410	68.7	—	—	9,633	60.0	59.4	1,527	71.5	80.1	366	55.2	44.6	675	57.2	50.5	156	82.7	88.2	1,755	84.6	90.1
JX5-c10087	10,087	75.1	—	—	—	—	—	1,527	72.4	81.7	366	56.0	46.4	675	58.8	50.9	156	76.3	86.3	1,755	81.3	88.0
JX5-c10084	10,084	75.8	—	—	—	—	—	1,527	71.2	79.9	366	52.5	41.7	768	56.4	56.3	156	82.1	88.2	1,755	83.8	90.1

**Variants**	**ORF6 (HSP90h)**			**ORF7 (p30)**			**ORF8 (CP)**			**ORF9 (p19)**			**ORF10 (p8)**			**ORF11**			**3′UTR**			
							
	**nt**	**nt%**	**aa%**	**nt**	**nt%**	**aa%**	**nt**	**nt%**	**aa%**	**nt**	**nt%**	**aa%**	**nt**	**nt%**	**aa%**	**nt**	**nt%**	**aa%**	**nt**	**nt%**		

**K75**	1,524		—	741		–	732			474			213			405			404			
Z2	1,524	83.0	87.2	741	76.3	69.9	732	83.5	89.7	474	85.0	86.6	213	81.2	72.9	—	—	—	—	—		
**JS27**	1,521	89.7	93.1	744	86.4	81.4	732	90.0	95.9	474	89.9	94.3	216	89.8	84.5	576	62.6	85.1	253	87.8		
**WH4-2**	1,524	88.7	92.7	741	86.5	80.5	732	88.3	93.4	474	88.6	93.6	216	88.4	81.7	570	82.5	82.1	252	88.1		
WH4-1	1,524	88.9	93.1	747	81.3	78.5	732	83.1	89.3	474	87.1	88.5	207	77.9	71.4	576	78.8	82.1	252	80.0		
CQ-c13707	—	—	—	—	—	—	—	—	—	—	—	—	—	—	—	—	—	—	—	—		
JX-c18869	1,524	83.7	88.8	741	80.6	76.4	732	90.0	90.5	474	83.5	86.0	216	81.0	74.6	576	80.7	81.3	—	—		
JX-c12007	1,524	89.6	93.7	738	87.8	83.7	732	88.1	93.8	474	88.6	91.1	213	91.1	87.1	570	86.7	88.1	—	—		
JX5-c18818	1,524	89.4	92.9	741	86.6	82.9	732	89.9	94.7	474	87.1	91.7	213	85.5	77.1	570	86.4	85.1	—	—		
JX5-c18410	1,524	83.7	88.6	741	80.0	76.0	732	86.5	90.1	474	81.7	84.7	216	79.6	75.7	—	—	—	—	—		
JX5-c10087	1,524	83.3	87.0	741	76.4	71.1	732	83.2	89.7	474	85.9	88.5	213	82.2	72.9	570	81.0	79.1	—	—		
JX5-c10084	1,524	83.6	89.0	741	82.1	77.6	732	86.2	92.6	474	82.1	86.6	216	80.1	69.0	570	81.2	79.9	—	—		

*“—” indicates partial or unavailable sequences of the virus genome. AcV-1 variants with full-length genome sequences are in bold font.*

Sequence alignment for each ORF (Table 2) encoded in the above genomic and contig sequences showed that sizes of ORF5, ORF6, ORF8, and ORF9 of these Chinese AcV-1 variants were the same as the corresponding ORFs of K75, except for the ORF6 in variant JS27. The ORF1b size of these Chinese AcV-1 variants was the same (1,527 nt), but 6-nt shorter than that of K75. The sizes of other ORFs were variable among these sequences. The ORF1a sizes showed the highest variation, ranging from 9,543 to 9,666 nt. ORF7 and ORF10 had three different sizes, and ORFs 2, 3, 4, and 11 had two sizes. Moreover, ORF11 sequences determined in this study were 165 nt or 171 nt larger than the ORF11 (405 nt) of K75 due to 6-nt and 164-nt deletions located near the 5′ and 3′ termini of ORF11 of K75, respectively (Supplementary Figure S1).

**FIGURE 2:**
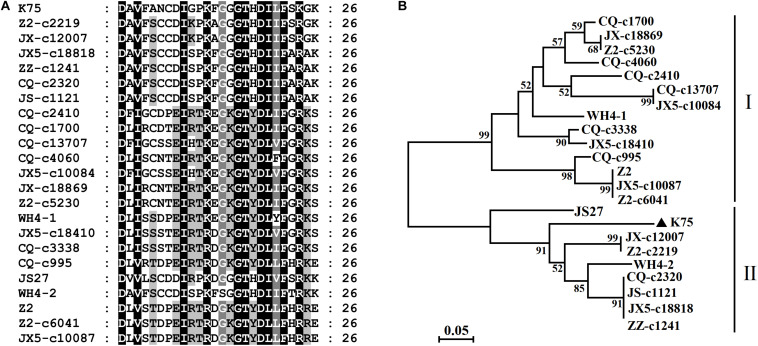
Multiple alignment of the 26-aa insert in RdRp of *Actinidia* virus 1 (AcV-1) variants **(A)** and a neighbor-joining phylogenetic tree inferred from the 26–amino acid (aa) insert sequences **(B)**.

High nucleotide and amino acid sequence diversity occurred in each ORF of the Chinese AcV-1 populations (Table 2). Generally, ORF1a, ORF2, and ORF3 near the 5′ terminus of AcV-1 genome were more variable. ORF1a of Chinese AcV-1 variants shared 59.5–86.1% nt sequence identity with each other and 60.0–73.2% nt sequence identity with the ORF1a of K75. RdRp encoded in ORF1b of all AcV-1 variants had an AcV-1 specific 26-amino acid (aa) insert located immediately after the GDD motif, when it was compared to RdRp encoded by other viruses in the family Closteroviridae. Multiple alignments showed that the 26-aa insertion sequences were highly variable among AcV-1 variants (Figure 2A) and formed two phylogenetic clades (Figure 2B). The ORF2 sequences of AcV-1 variants determined in this study were even more variable and showed the highest divergence up to 47.5% at nt sequence level and 58.9% at aa sequence level compared to isolate K75. Nucleotide variations distributed across the entire ORF2 (Supplementary Figure S2A) and amino acid variations were mainly present at the C-terminal half of the encoded protein (p13) (Supplementary Figure S2B). Similarly, the ORF3 of these Chinese AcV-1 variants shared 56.2–86.4% nt and 50.5–85.7% aa sequence identity with that of K75. ORFs 5, 6, 8, and 9 were relatively conserved among AcV-1 variants by showing greater than 80% nt sequence identity with the corresponding sequences of K75 (Table 2).

**FIGURE 3:**
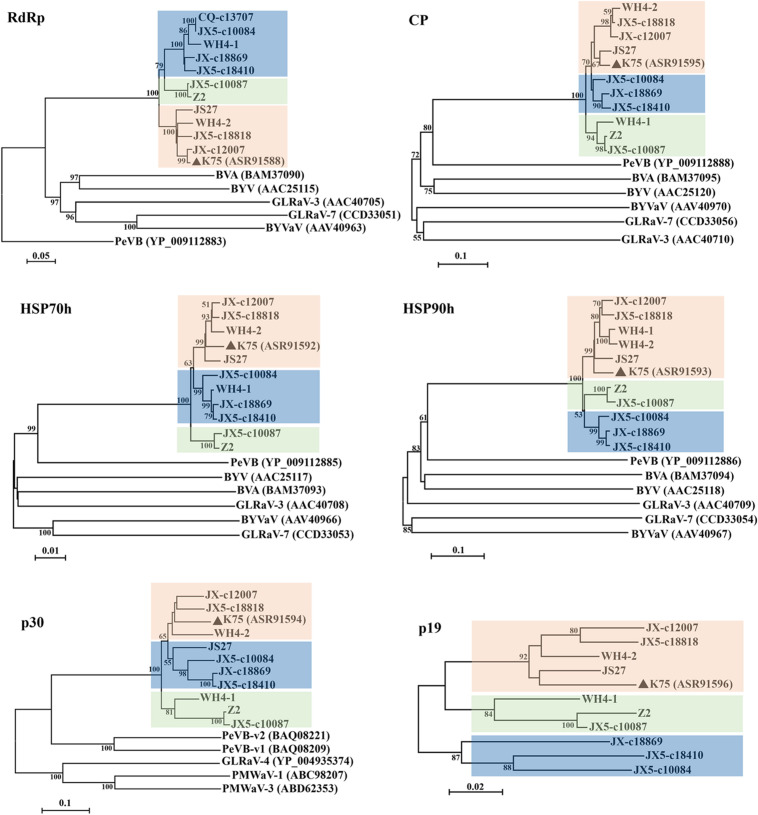
Phylogenetic trees constructed using amino acid (aa) sequences of putative RdRp, CP, HSP70h, HSP90h, p30, and p19 of *Actinidia* virus 1 (AcV-1) variants. The genomic sequence of AcV-1 isolate K75, two complete (JS27 and WH4-2) and two near-complete (Z2 and WH4-1) genomic sequences determined in this study, and seven RNA-seq derived contigs with sizes > 10,000 nt, were included in the analysis. The contig CQ-c13707 covering the ORF1a-ORF4 (position 248–14,118 nt) of the viral genome was only included in the RdRp-based tree. Bootstrap values (1,000 replicates) > 50% were shown at branch nodes. The reported AcV-1 isolate K75 was marked by a black triangle. GenBank accession numbers of viruses referred for phylogenetic analyses were indicated following the abbreviation of each virus name. PeVB, Persimmon virus B (AB923924); BVA, blueberry virus A (AB733585); BYV, beet yellows virus (AF056575); BYVaV, blackberry yellow vein-associated virus (AY776334); GLRaV-3, grapevine leafroll-associated virus 3 (AF037268); GLRaV-7, grapevine leafroll-associated virus 7 (HE588185).

Phylogenetic analyses for the complete aa sequences of proteins RdRp, HSP70h, and CP, for which aa sequences are specified by ICTV for genus demarcation within the family Closteroviridae, showed that all Chinese AcV-1 variants together with isolate K75 clustered into a clade, distantly related to persimmon virus B (PeVB), an unassigned closterovirus species (Ito et al., 2015) (Figure 3). In the AcV-1 clade, Chinese AcV-1 variants formed three subclades represented by JX5-c18410, Z2, and K75. In the HSP90h-, p30-, and p19-based trees, AcV-1 variants also clustered into three clades. It was found that the positions of some AcV-1 variants changed in these phylogenetic trees. For example, in the RdRp- and HSP70h-based trees, WH4-1 was in the subclade represented by JX5-c18410, but clustered in the subclade represented by K75 in the HSP90h-based tree and in the subclade represented by Z2 in the CP-, p30-, and p19-based trees.

### Identification of Domains of AcV-1 Proteins

**FIGURE 4:**
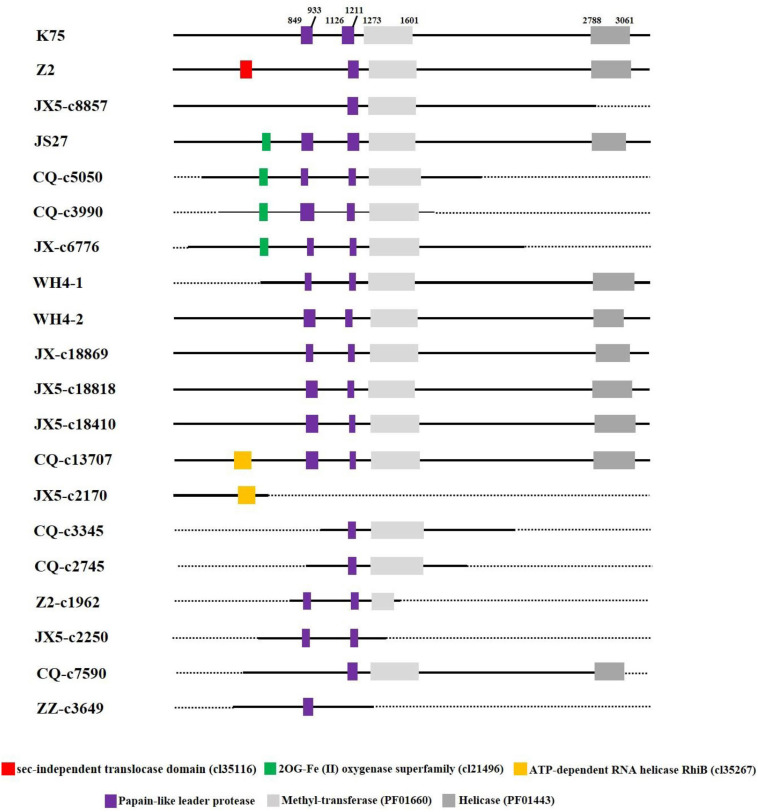
Schematic diagram of domains contained in ORF1a of *Actinidia* virus 1 (AcV-1) variants. The starting and ending sites of each domain in the ORF1a of AcV-1 isolate K75 are indicated by black numbers. The sec-independent translocase domain (cl35116), domain belonging to the 2OG-Fe (II) oxygenase superfamily (cl21496), and ATP-dependent RNA helicase RhiB (cl35267) are marked as red, green, and orange boxes, respectively. MET and HEL are shown as light gray and dark gray boxes, respectively. Black solid lines represent available sequences, and black dotted lines indicate unavailable sequences.

Putative domains of the proteins predicted from AcV-1 genomic sequences (including assembled contig sequences) were identified by using CDD at https://www.ncbi.nlm.nih.gov/Structure/cdd. It was found that motifs of papain-like leader protease (L-Pro), methyltransferase (MTR, PF01660), and helicase (HEL, PF01443), necessary for virus replication, were present in the multifunctional protein encoded in ORF1a of all AcV-1 variants (Figure 4). However, the number of L-Pro domains varied among AcV-1 variants. Notably, the ORF1a of Z2, JX5-c8857, CQ-c7590, and ZZ-c3649 had a single L-Pro domain (L1 or L2), and the ORF1a of other AcV-1 variants had two L-Pro domains (L1 and L2). Sequence analyses revealed the presence of a sec-independent translocase domain (cl35116) in variant Z2 and an ATP-dependent RNA helicase RhiB (cl35267) in variants CQ-c13707 and JX5-c2170. These domains were not identified and documented in the reported AcV-1 isolate K75. Further sequence alignments showed that the sequences covering the two domains were highly variable in other AcV-1 variants (Supplementary Figures S3A,B). Additionally, according to the CDD prediction, ORF1a of the AcV-1 variants JS27, CQ-c5050, CQ-c3990, and JX-c6776 harbored a domain (56 or 57 aa) of 2OG-Fe (II) oxygenase superfamily (cl21496) (Aravind and Koonin, 2001; Erwin et al., 2008) upstream of the MET domain. Further alignment showed that all AcV-1 variants had the core sequence of AlkB domain (pfam 03171) (Maree et al., 2008; Ghanem-Sabanadzovic et al., 2012; Donda et al., 2017), belonging to 2OG-Fe (II) oxygenase superfamily, as in some closteroviruses (Supplementary Figure S3C).

A thaumatin-like domain was identified in protein p30 encoded by ORF7 of all available AcV-1 variants. The thaumatin-like domain of all available AcV-1 variants was 109 or 115 aa (Supplementary Figure S4) and shared 69.9–96.3% aa sequence identity with each other.

### Recombination Analysis

The incongruent positions of some variants in phylogenetic trees based on the sequences of different proteins indicated possible recombination events occurring in the Chinese AcV-1 variants. Recombination analysis performed on an alignment of 11 AcV-1 genomic sequences revealed that 11 recombination events were present in eight variants (Table 3). All the detected recombination regions located at the 3′ half of the viral genome. Three very close recombination events at positions 13,029–14,361, 14,881–16,595, and 16,614–18,703 nt were detected in WH4-1 with JX5-c18410 as a possible major parent in two recombination events (events 2 and 3) and as a possible minor parent in one recombination event (event 1). The variant WH4-2 from the same sample as WH4-1 had two recombination events (events 4 and 5) with parents different from those of WH4-1. It was found that three recombination events (events 4, 8, and 10) in variants WH4-2, Z2, and JX5-c10087 had a common major parent JX5-c18818. Additionally, two variants JX-c12007 and JS27, for which the start sites of recombination were not detected, had the same parents WH4-2 and K75. AcV-1 variants JX5-c18410, JX5-c10087, and JX5-c10084 were from the same sample (ID: JX5), but had different parents.

**TABLE 3:** Putative recombination events identified by using RDP4 in *Actinidia* virus 1 (AcV-1) genomic sequences.

Event	Recombinant	Parent		Break point		Av. *P*-value						
				
		Major	Minor	Start	End	R	G	B	M	C	S	P
1	WH4-1	JX5-c10084	JX5-c18410	13,029	14,361	3.519E-16	1.179E-17	3.952E-16	2.231E-11	4.889E-09	4.236E-20	2.300 E-05
2	WH4-1	JX5-c18410	WH4-2	14,881	16,595	1.200E-127	6.732E-146	2.192E-139	9.358E-25	5.854E-15	2.408E-35	2.198E-13
3	WH4-1	JX5-c18410	Z2	16,614	18,703	3.609E-51	—	1.973E-58	4.951E-22	4.348E-10	1.262E-18	—
4	WH4-2	JX5-c18818	K75	11,310	11,534	7.772E-09	—	6.056E-10	2.336E-05	2.050E-06	—	2.777E-07
5	WH4-2	JX-c12007	JX5-c10084	18,618	18,834	6.659E-03	—	—	2.484E-05	3.751E-03	1.461E-06	1.059E-03
6	JS27	K75	WH4-2	—	11,078	3.448E-20	—	7.854E-10	2.855E-19	3.342E-25	3.110E-92	2.442E-14
7	JX-c12007	WH4-2	K75	—	11,518	5.338E-51	3.235E-42	2.153E-43	4.727E-24	2.318E-10	1.618E-75	8.976E-09
8	Z2	JX5-c18818	WH4-1	11,866	13,102	1.596E-16	1.544E-02	1.265E-12	7.382E-08	2.315E-09	3.237E-16	—
9	JX5-c18410	JX-c18869	JX5-c10084	17,834	18,245	1.278E-06	—	7.447E-07	4.517E-07	5.658E-08	—	1.697E-06
10	JX5-c10087	JX5-c18818	Unknown	13,129	18,237	—	—	—	2.539E-09	9.841E-05	9.409E-06	1.580E-05
11	JX5-c10084	WH4-1	Unknown	11,468	12,506	3.903E-04	—	3.443E-03	2.431E-03	1.946E-04	1.581E-10	—

*“—” indicates that no recombination event was predicted.*

### RT-PCR Detection of AcV-1 in Kiwifruit Plants Grown in China

To disclose the infection status of AcV-1, leaf samples collected from 245 kiwifruit plants grown in 10 provinces and cities in China were subjected to RT-PCR detection of AcV-1 using the primer set cp-F/cp-R. Results showed that 75 samples, including 70 symptomatic samples and five asymptomatic samples, were positive for AcV-1 (Table 4), accounting for 30.6% of total samples. The virus was detected in four analyzed species, including *A. chinensis*, *A. delicious*, *A. arguta*, and *A. eriantha*, and in some other kiwifruit plants with species unknown. Of these AcV-1–positive samples, two samples ZZ4 and CQ11 were negative for other detected viruses, and 73 samples were coinfected by AcV-1 with one or more other viruses, including AcVA, AcVB, ASGV, CLBV, and AcCRaV (Supplementary Table S4), and/or unreported kiwifruit-infecting viruses (data not shown). Except for samples from Yunnan Province and Shanghai city, where all collected samples were negative for AcV-1 in this test, the virus was detected in samples from seven provinces and one city, with relative high infection rates for samples from Hubei and Shandong provinces and Chongqing city (Table 4).

**TABLE 4:** Incidence of *Actinidia* virus 1 (AcV-1) in the kiwifruit samples collected from eight provinces and two cities in China.

Origin	No. of Samples	Infected/tested (%)	
		
		Symptomatic^a^	Asymptomatic
Hubei	71	27/63 (42.9)	3/8
Jiangxi	14	1/13 (7.7)	1/1
Shandong	28	16/28 (57.1)	0/0
Zhejiang	31	4/29 (13.8)	0/2
Henan	32	4/32 (12.5)	0/0
Fujian	7	1/6 (16.7)	0/1
Shanxi	4	3/4 (75)	0/0
Chongqing	36	14/34 (41.2)	1/2
Yunnan	20	0/20	0/0
Shanghai	2	0/2	0/0
Total	245	70/231 (30.3)	5/14 (35.7)

**^*a*^The symptoms of the tested samples include uneven chlorosis, mosaic, ringspot, chlorotic spot, and deformation.**

The 376 bp PCR products obtained using the primer set cp-F/cp-R from 75 AcV-1–positive samples were sequenced. These sequences together with the corresponding sequences derived from RNA-seq and 13 sequences of CP gene referred from GenBank database were used for phylogenetic analysis. The 376-bp sequences of all Chinese AcV-1 variants showed 78.5–100% nt identity with the equivalent sequence of isolate K75 and clustered into three distinct clades, respectively, represented by the variants K75, WH4-1, and Z2, except for one variant YT5, which was distant from the three clades (Supplementary Figure S5). All eight AcV-1 variants, previously reported from Sichuan province (Peng et al., 2018), clustered in the same clade (clade I). It was found that variants from each of samples Z2 and JX5 were distributed in different clades, confirming the sequence variation within the AcV-1 populations.

## Discussion

NGS combined with bioinformatics analysis has become a routine technology for the rapid discovery and characterization of known or novel plant viruses (Adams et al., 2009; Kreuze et al., 2009; Barba et al., 2014; Wu Q. et al., 2015; Hadidi et al., 2016; Cretazzo and Velasco, 2017; Villamor et al., 2019; Zhao et al., 2020; Wang et al., 2020; Zhang et al., 2020). NGS-based metagenomic analyses have revealed the natural biodiversity of plant viruses (Roossinck, 2011; Roossinck et al., 2015). In this study, the RNA-seq analyses of two samples individually collected from two kiwifruit plants and four pooled kiwifruit samples revealed the presence of the eight kiwifruit-infecting viruses AcV-1, AcCRaV, ASbLV, AcVA, AcVB, CLBV, AcEV-2, and ASGV, and three viruses related to the families Bromoviridae, Betaflexiviridae, and Secoviridae. These data indicate that NGS technology is useful for identifying viruses infecting kiwifruit plants.

AcV-1 was identified from all six samples analyzed by RNA-seq. Except for the sample ZZ, from which AcV-1 contigs with size of 224–4,132 nt covering 71.5% of the viral genome were recovered, large contig sequences (>5,000 nt) of AcV-1 genome were obtained from the remaining five RNA-seq samples, indicating that the RNA-seq analyses were efficient for the genomic and molecular characterization of the virus. The RNA-seq sample ZZ was a pool of leaf samples from 30 kiwifruit plants, of which only two were positive for AcV-1. The relatively low AcV-1 titer in the mixed sample ZZ and molecular diversity of the virus in the two sampled plants might contribute to the absence of large contig sequences from the sample. The Sanger sequencing for the complete genome of AcV-1 variants JS27 and Z2 also confirmed the reliability of NGS sequence assembly for the viral genome. RNA-seq analyses also revealed mixed infection of AcV-1 variants in its natural host. Two contigs covering nearly the complete genome of AcV-1 were assembled from the sample JX5 and shared about 63% nt sequence identity, and the remaining contigs from the sample shared less than 85% nt sequence identity with each other. The constitution of AcV-1 contigs from sample Z2 was even more complicated. From the sample, 26 AcV-1 contigs were derived, but could not be assembled as contiguous sequences, indicating high sequence heterogeneity of these AcV-1 contigs, which was also the case in the pooled sample CQ. Consistent with RNA-seq analyses, Sanger sequencing for RT-PCR products revealed at least two divergent AcV-1 variants (WH4-1 and WH4-2) in the sample WH4. Like viruses infecting other perennial woody plants, viruses can be transmitted among kiwifruit plants through vegetative propagation. Vectors play important roles in the natural transmission of viruses in the family Closteroviridae, which might increase the virus diversity in a single host plant (Iglesias et al., 2008; Roy and Brlansky, 2009; Rubio et al., 2013).

The complete genomic sequences of variants JS27 and WH4-2 determined here were 18,896 and 18,851 nt, respectively, close to the 18,848 nt of reported isolate K75 (Blouin et al., 2018). However, the 3′-UTR of JS27, WH4-2, and a partially sequenced variant WH4-1 was about 150 nt shorter than that of K75. Analyses of the two complete genomic sequences of AcV-1 variants JS27 and WH4-2 and nine near-complete genomic sequences of AcV-1 variants Z2, WH4-1, CQ-c13707, JX-c18869, JX-c12007, JX5-c18818, JX5-c18410, JX5-c10087, and JX5-c10084 derived from different kiwifruit plants and geographical areas in China revealed high genetic divergence across the viral genome, especially in the 5′-UTR and the ORFs (ORF1a, ORF2, and ORF3) located in the 5′ half of the viral genome. The sequence variation might impede primer design to amplify the viral 5′ terminal sequence of variant WH4-1. The 5′ UTR could play a regulatory role in virus replication (Mawassi et al., 1996; Liveratos et al., 2004; Mongkolsiriwattana et al., 2016; Jarugula et al., 2018). The 5′ UTR of GLRaV-3 contains critical elements required for virus replication (Jarugula et al., 2010; Jarugula et al., 2018; Adiputra et al., 2019). Similarly, the 5′ UTR of CTV is also highly variable (Lopez et al., 1998), but can form stable SL (stem-loop) structures, which are essential for viral replication and assembly (Tatineni et al., 2002; Gowda et al., 2003).

The ORF1a of viruses in the family Closteroviridae encodes a multifunctional protein (Rubio et al., 2013). The size of ORF1a differed among all available AcV-1 variants with divergence up to 40% at nt sequence level. Previously, the ORF1a size variation was also found in GLRaV-2 (Meng et al., 2005) and GLRaV-5 (Thompson et al., 2012). Overall, the organization of ORF1a of the AcV-1 genome is similar to that of members in the family Closteroviridae (Agranovsky et al., 1995; Karasev, 2000; Dolja et al., 2006). In this study, we found that AcV-1 variants might have one or two L-Pro domains (L1 and L2). Some viruses in the family Closteroviridae possess a tandem of papain-like cysteine proteases with two divergent functional domains L1 and L2, which might have evolved via gene duplication (Karasev, 2000; Peng et al., 2001; Dolja et al., 2006; Liu et al., 2009). The absence of domain L1 in some AcV-1 variants indicated that the domain L2 might be indispensable, which was different from the case that domain L1 plays a crucial role in the establishment of infection and accumulation of some viruses in the family Closteroviridae (Liu et al., 2009; Kang et al., 2018). Except for motifs L-Pro, MTR, and HEL, necessary for viral replication, the ORF1a of some AcV-1 variants harbors a sec-independent translocase domain, or an ATP-dependent RNA helicase RhiB, or an 2OG-Fe (II) oxygenase domain, suggesting that these domains might have been acquired relatively recently via horizontal gene transfer (Bratlie and Drablos, 2005). The AlkB domain is present in the polyproteins of some viruses in the family Closteroviridae, as well as in alpha-like plant viruses belonging to genera *Allexivirus*, *Carlavirus*, *Foveavirus*, *Potexvirus*, *Trichovirus*, and *Vitivirus* (Erwin et al., 2008). The AlkB domain usually locates between MET and HEL domains, whereas the AlkB domain of AcV-1 variants identified in this study locates upstream of the MET domain. Certain cellular AlkBs are involved in RNA repair via methylation reversal (Aravind and Koonin, 2001). However, the function of AlkB in plant virus infection is unknown. The variation of L-Pro numbers and other domains in the multifunctional protein might show not only the diversity of the AcV-1 populations, but also different biological features (Liu et al., 2009; Atallah et al., 2016; Kang et al., 2018). The p30 encoded in AcV-1 ORF7 has a thaumatin-like domain like that in the p29 of PeVB-v1 (GenBank accession no. AB923924) and the p28 of PeVB-v2 (GenBank accession no. AB923925). Although the location of p30 in the AcV-1 genome is similar to the CPm of some closteroviruses, the p30 did not show aa sequence identity with the CPm encoded by other viruses in the family Closteroviridae. The function of the protein remains to be unknown.

The ORF-by-ORF comparisons also showed that the sequences of ORF2 and ORF3 were highly variable among the AcV-1 variants, with the highest divergence up to 58 and 49% at aa sequence level, respectively. Similar diversities are observed among divergent variants of some closteroviruses, such as GLRaV-3 (Maree et al., 2008), GLRaV-4 (Adiputra et al., 2019), and PeVB (Ito et al., 2015). However, other ORFs were much more conserved in AcV-1 populations. Among the viral variants, the aa divergence values of 10–20% for CP, HSP70h and RdRp are less than the species discriminating threshold of 25% approved by ICTV. Therefore, we contend that all AcV-1 sequences represent divergent variants of the virus, which often occurred in a single kiwifruit plant as illustrated by RNA-seq analyses. Phylogenetic analyses for the complete aa sequences of proteins RdRp, HSP70h and CP showed that all Chinese AcV-1 variants together with isolate K75 clustered into a clade, distantly related to PeVB. In the AcV-1 clade, Chinese AcV-1 variants formed three subclades. The results further support that the sequences determined from kiwifruit plants grown in China belong to AcV-1 variants, which are highly divergent in molecular composition.

Recombination in RNA viruses has been extensively documented as a powerful driving force for generating new variants (Karasev, 2000; Chare and Holmes, 2006; Farooq et al., 2012; Katsiani et al., 2015; James and Phelan, 2016; Ruiz et al., 2018). Frequent homologous recombination events have been reported for viruses in the family Closteroviridae (Karasev, 2000; Turturo et al., 2005; Sztuba-Solińska et al., 2011; Rubio et al., 2013). The incongruent positions of some AcV-1 variants in the phylograms based on proteins coded near the 3′ half of AcV-1 genome suggested occurrences of recombination events. Although the parents for these recombinants were identified, some parents were also potential recombinants. The exact parents for these recombinants need to be clarified as more genomic sequences available from a wide host source. The data will help to understand whether an ancient recombination event occurred before the recombinants spread worldwide, as previously suggested for CTV (Roy et al., 2005; Rubio et al., 2013; Wu G. et al., 2015). Most of the recombination events occurred in the region following the viral ORF1b. In CTV, the genes encoded in the region play important roles in the determination of pathogenicity, movement, and host range (Folimonova, 2020). The frequent recombination events in the region of AcV-1 genome might be necessary to meet possible selection pressure (Roy and Brlansky, 2009).

Finally, RT-PCR detection showed that AcV-1 occurred in four *Actinidia* species and some kiwifruit plants of unknown species grown in seven provinces and one city in China. Previous investigation showed that mixed infection of several viruses in a single kiwifruit plant was very common (Zheng et al., 2014, 2017; Blouin et al., 2018; Veerakone et al., 2018; Wang et al., 2020). Most of the plants tested in this study showed viral disease–like symptoms, including uneven chlorosis between veins, mosaic, ringspots, chlorotic spots, and/or deformation, which were indicated previously to be associated with some virus infections. However, some asymptomatic plants were also positive for the virus. Although two AcV-1–infected samples ZZ4 and CQ11 exhibited interveinal chlorosis and were negative for other kiwifruit viruses known in China, we could not conclude that the disease symptom was caused by the virus infection due to the potential infection of unknown viruses. Therefore, the association of AcV-1 infection with diseases of kiwifruit plants is unclear at present. The biological properties of AcV-1 need to be determined in future studies.

All nucleotide sequences obtained in this study supported high molecular diversity in AcV-1 populations from kiwifruit plants grown in China. This represents the first report for the complete genome sequences of AcV-1 in China and molecular diversity of AcV-1. The reported AcV-1 sequences would be helpful for further detailed taxonomic study and assignment of efficient molecular diagnostic techniques of AcV-1 to improve the sanitary status of kiwifruit planting materials in China.

## Data Availability Statement

The datasets presented in this study can be found in online repositories. The names of the repository/repositories and accession number(s) can be found in the article/Supplementary Material.

## Author Contributions

NH and GW supervised the project, conceived and designed the experiments. SW, ZY, MR, QL, and YW performed the experiments and analyzed the data. SW and NH wrote the manuscript, prepared the tables and figures, revised and approved final version of the manuscript. This is the first submission of the manuscript and it is not being considered for publication elsewhere in part or in whole. All authors approved the submission of this manuscript.

## Conflict of Interest

The authors declare that the research was conducted in the absence of any commercial or financial relationships that could be construed as a potential conflict of interest.

## Footnotes

1 http://www.ncbi.nlm.nih.gov/

2 https://www.ncbi.nlm.nih.gov/Structure/cdd/wrpsb.cgi

3 http://www.ebi.ac.uk/Tools/msa/clustalw2/

## Supplementary Material

Supplementary Figure 1 Multiple alignment of nucleotide sequences of ORF11 of Chinese Actinidia virus 1 (AcV-1) variants and reported isolate K75.

Supplementary Figure 2 Multiple alignment of nucleotide **(A)** and amino acid **(B)** sequences of ORF2 of Actinidia virus 1 (AcV-1) variants.

Supplementary Figure 3 Amino acid sequence alignments of sec-independent translocase domain **(A)**, ATP-dependent RNA helicase RhiB domain **(B)** and AlkB domain belonging to the 2OG-Fe (II) oxygenase superfamily **(C)** of Actinidia virus 1 (AcV-1) variants or viruses. Black triangle indicates the AcV-1 variants containing predicated domains. A solid line above aligned sequences marks the core of the AlkB domain and arrows indicate the conserved residues in the AlkB. Accession numbers of sequences used in the analysis include: grapevine leafroll-associated virus 1 (GLRaV-1) isolates WA-CH (KU674796), WA-PN (KU674797) and Canada (NC_016509), GLRaV-3 (GU983863), GLRaV-4 (FJ467503), GLRaV-5 (JX559640), GLRaV-6 (NC_016417), GLRaV-9 (AY297819), little cherry virus 2 (LChV-2; AF531505).

Supplementary Figure 4 Multiple sequence alignment of thaumatin-like domain in ORF7 (p30) of the Actinidia virus 1 (AcV-1) variants.

Supplementary Figure 5 Neighbor joining (NJ) phylogenetic tree generated from the nucleotide sequences of partial CP of Actinidia virus 1 (AcV-1) variants. Bootstrap values (1,000 replicates) > 50% are shown at branch nodes. The reported AcV-1 isolate K75 is marked by a black triangle. The sequences referred from GenBank are identified by their GenBank accession numbers. The scale bar is 0.01 substitutions per site.
