# Supplementary figures and images for: Next-Generation Sequencing Combined With Conventional Sanger Sequencing Reveals High Molecular Diversity in Actinidia Virus 1 Populations From Kiwifruit Grown in China
Source: Front Microbiol. 2020 Dec 16;11:602039. doi: 10.3389/fmicb.2020.602039 (PMC7774462; doi:10.3389/fmicb.2020.602039)

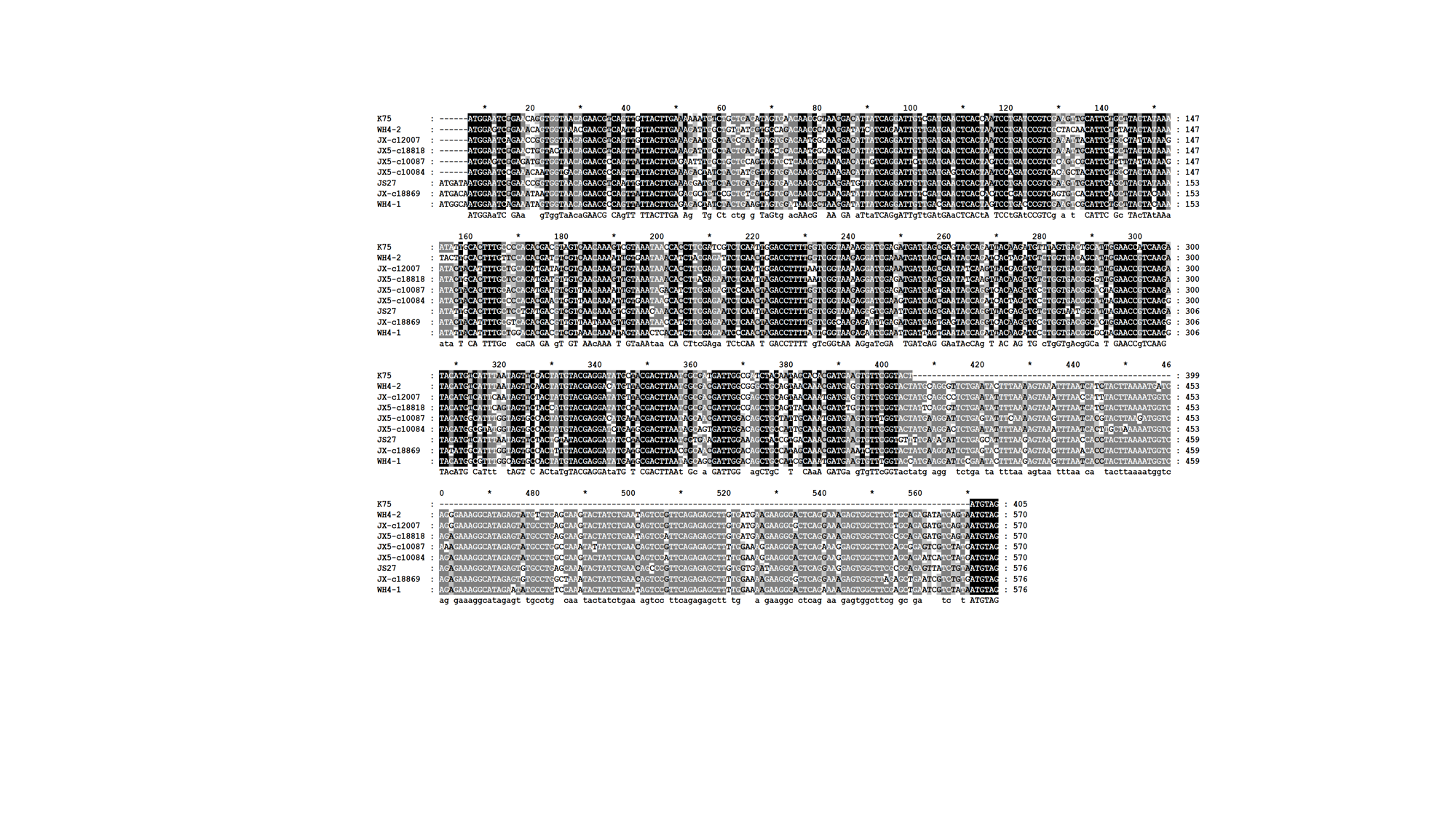

Supplement: Supplementary Figure 1 — Multiple alignment of nucleotide sequences of ORF11 of Chinese Actinidia virus 1 (AcV-1) variants and reported isolate K75. [file Image_1.TIF]

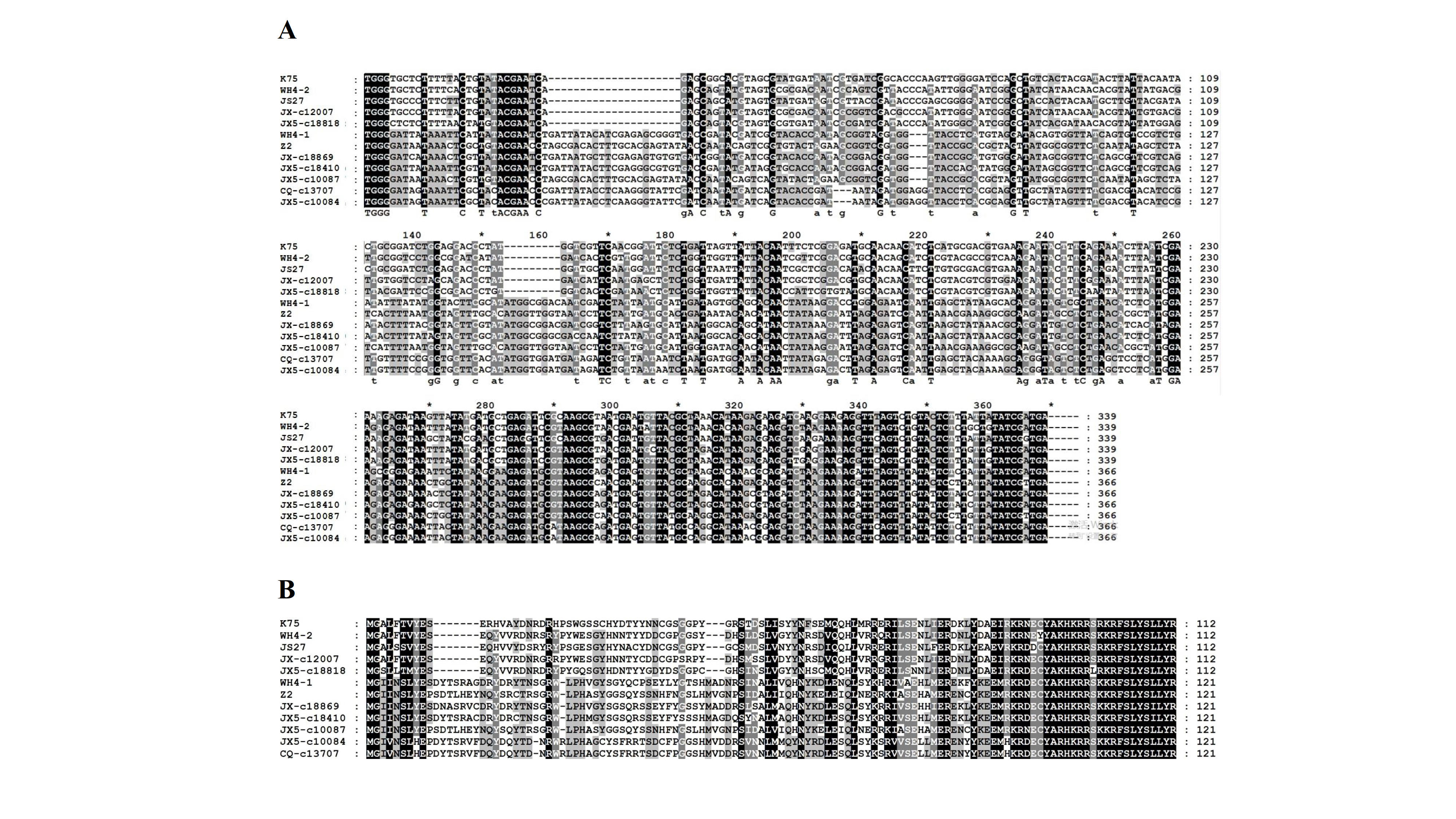

Supplement: Supplementary Figure 2 — Multiple alignment of nucleotide (A) and amino acid (B) sequences of ORF2 of Actinidia virus 1 (AcV-1) variants. [file Image_2.JPEG]

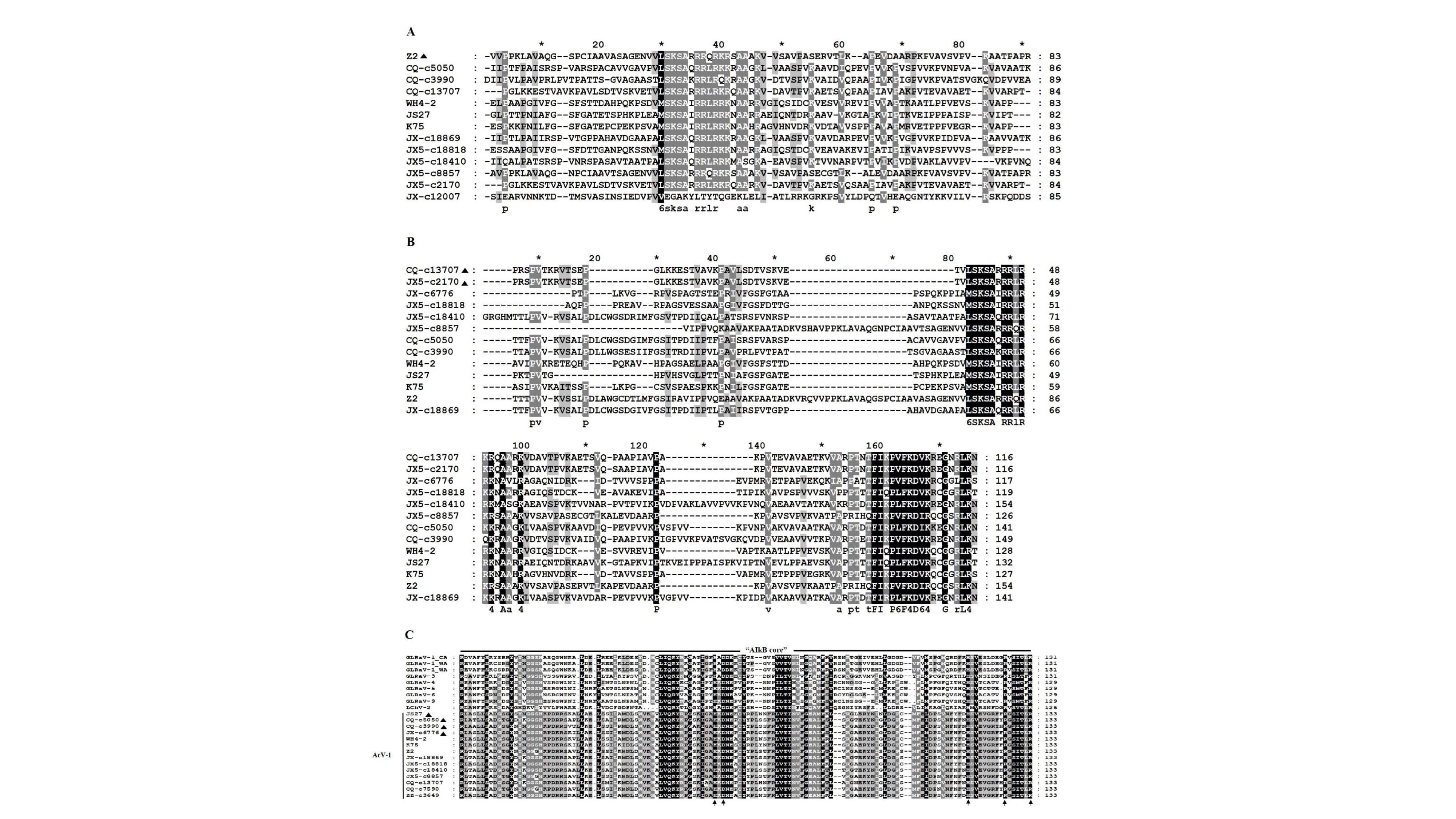

Supplement: Supplementary Figure 3 — Amino acid sequence alignments of sec-independent translocase domain (A), ATP-dependent RNA helicase RhiB domain (B) and AlkB domain belonging to the 2OG-Fe (II) oxygenase superfamily (C) of Actinidia virus 1 (AcV-1) variants or viruses. Black triangle indicates the AcV-1 variants containing predicated domains. A solid line above aligned sequences marks the core of the AlkB domain and arrows indicate the conserved residues in the AlkB. Accession numbers of sequences used in the analysis include: grapevine leafroll-associated virus 1 (GLRaV-1) isolates WA-CH (KU674796), WA-PN (KU674797) and Canada (NC_016509), GLRaV-3 (GU983863), GLRaV-4 (FJ467503), GLRaV-5 (JX559640), GLRaV-6 (NC_016417), GLRaV-9 (AY297819), little cherry virus 2 (LChV-2; AF531505). [file Image_3.JPEG]

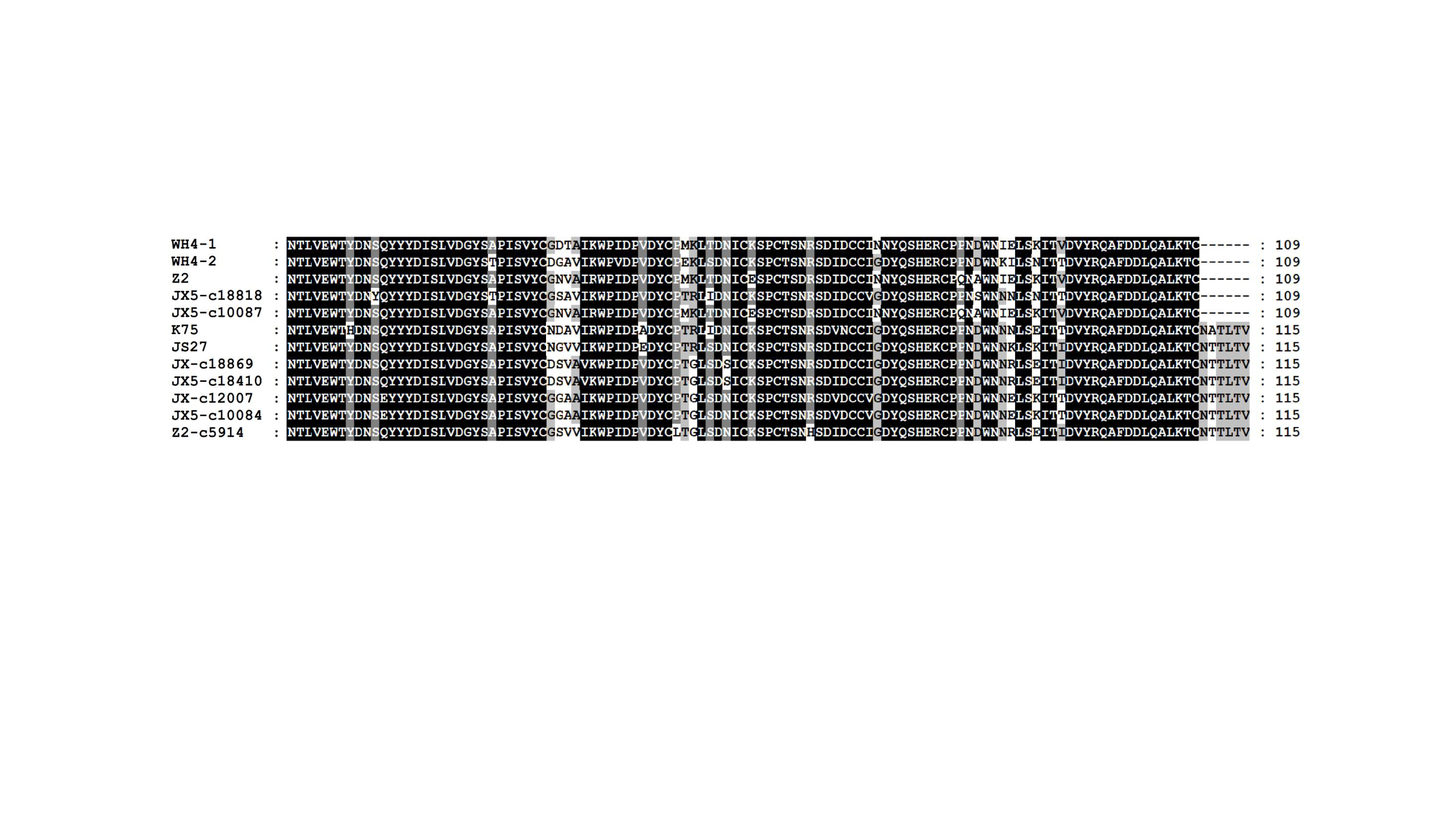

Supplement: Supplementary Figure 4 — Multiple sequence alignment of thaumatin-like domain in ORF7 (p30) of the Actinidia virus 1 (AcV-1) variants. [file Image_4.TIF]

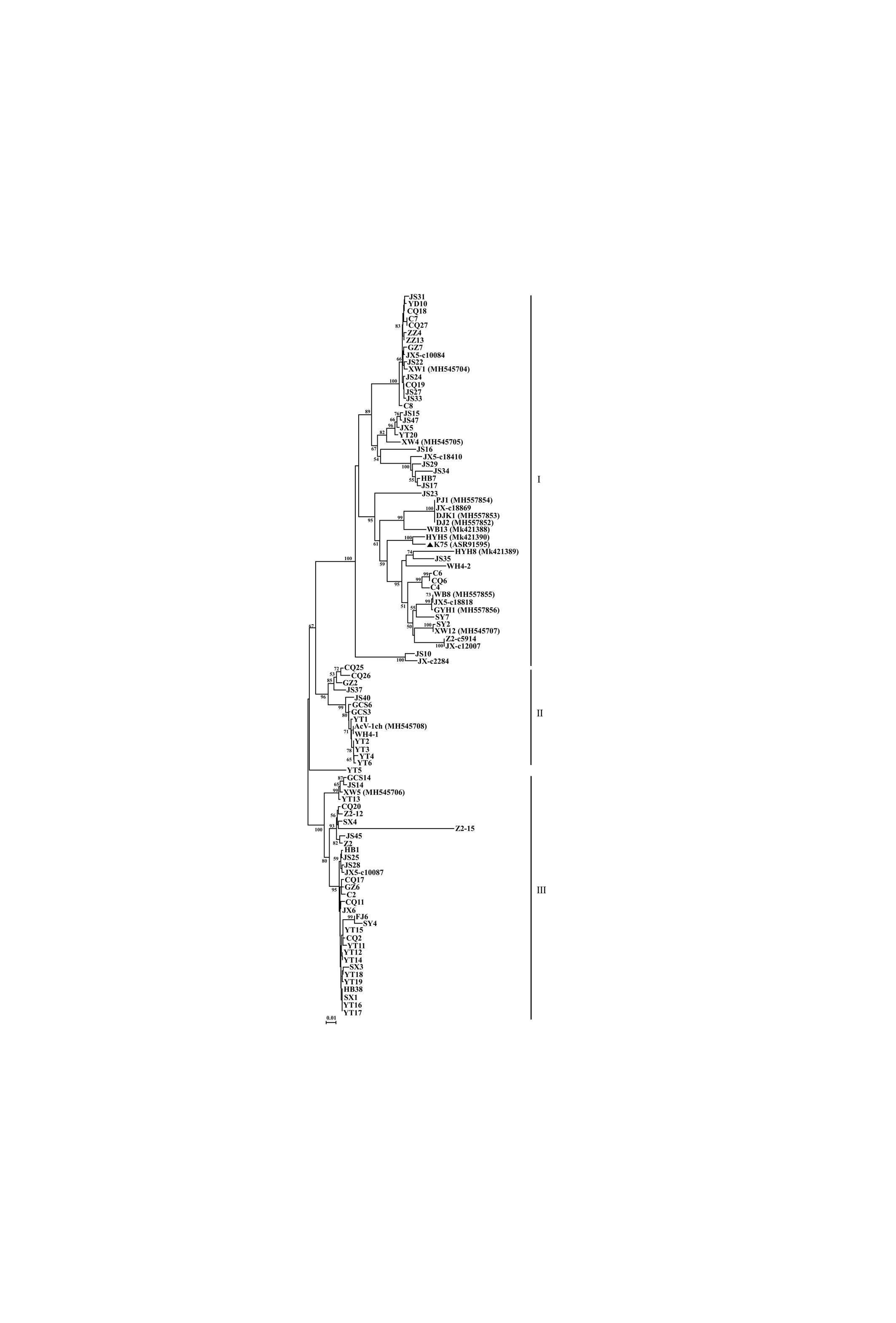

Supplement: Supplementary Figure 5 — Neighbor joining (NJ) phylogenetic tree generated from the nucleotide sequences of partial CP of Actinidia virus 1 (AcV-1) variants. Bootstrap values (1,000 replicates) > 50% are shown at branch nodes. The reported AcV-1 isolate K75 is marked by a black triangle. The sequences referred from GenBank are identified by their GenBank accession numbers. The scale bar is 0.01 substitutions per site. [file Image_5.TIF]
