# Supplementary material for: Next-Generation Sequencing Combined With Conventional Sanger Sequencing Reveals High Molecular Diversity in Actinidia Virus 1 Populations From Kiwifruit Grown in China
Source: Front Microbiol. 2020 Dec 16;11:602039. doi: 10.3389/fmicb.2020.602039 (PMC7774462; doi:10.3389/fmicb.2020.602039)
Supplement: Supplementary file 6 [file Data_Sheet_1.docx]

**TABLE S1** Primers used for the RT-PCR detection of AcCRaV, ASGV, AcV-1, CLBV, AcVA and AcVB

| **Virus** | **Primer** | **Sequence (5′- 3′)** | **Position (nt)** | **Product (bp)** | **Reference** |
| --- | --- | --- | --- | --- | --- |
| AcCRaV | 3F | ATCCAAGAATTCCTTAACAGCA | 683-704 | 477 | Zheng et al., 2017 |
|  | 3R | TGTGCAATCATGGCTTATCAGA | 1138-1159 |  |  |
| ASGV | 499F | CCCGCTGTTGGATTTGATACACCTC | 5873-5897 | 499 | James D, 1999 |
|  | 499R | GGAATTTCACACGACTCCTAACCCTCC | 6345-6371 |  |  |
| AcV-1 | CP-F | TGAGCTRGGRATAGATGTTGC | 16607-16627 | 375 | Wen et al., 2018 |
|  | CP-R | TCTCTCAGGGTTMGGATGAGT | 16962-16982 |  |  |
| CLBV | 1F | AGCCATAGTTGAACCATTCCTC | 7216-7237 | 453 | Harper et al., 2008 |
|  | 5R | GCATGTGGTGAATGATCTGC | 7650-7669 |  |  |
| AcVA | 5F | CATCATTTCTCACGGGTAGGA | 7120-7142 | 238 | Blouin et al., 2012 |
|  | 5R | TCACACAGACACTCCACACAG | 7383-7405 |  |  |
| AcVB | 5F | GTTTGCGAGGAGACGTAGGGC | 7118-7140 | 342 | Blouin et al., 2012 |
|  | 5R | AGTTAAGTGCTCTYGGRGGTGTG | 7438-7460 |  |  |

R: A/G, M: A/C, Y: T/C.

**TABLE S2** Origins of six RNA-seq samples, and the RNA-seq derived reads and assembled contigs

| Sample | Origin  (province/city) | Sample constitute | *Actinidia* spp. | Total Reads | Clean Reads | No. of total contig | | No. of viral contig |
| --- | --- | --- | --- | --- | --- | --- | --- | --- |
| JS | Hubei | JS27, JS29, JS30, JS45 | *A. chinensis;* unknown species | 68,653,600 | 67,640,546 | 177,848 | 254 | |
| ZZ | Henan | ZZ1-ZZ11, ZZ13- ZZ31 | *A. chinensis; A. delicious;*  *A. argute*; *A. eriantha*  *A. rufa*; unknown species | 93,183,834 | 92,477,928 | 315,308 | 154 | |
| Z2 | Henan | ZZ12 | *A. delicious* | 73,827,030 | 73,387,306 | 165,312 | 137 | |
| CQ | Chongqing | CQ1-CQ28 | *A. chinensis; A. delicious;*  *A. argute*; unknown species | 83,404,704 | 82,730,664 | 246,566 | 620 | |
| JX | Jiangxi | JX1-JX4, JX6 | *A. chinensis*; unknown species | 80,491,928 | 79,782,996 | 221,290 | 73 | |
| JX5 | Jiangxi | JX5 | *A. chinensis* | 74,066,216 | 73,419,336 | 174,235 | 18 | |

**TABLE S3** Primers used for the amplification of the genomic RNA of AcV-1

| **Variant** | **Primer** | **Sequence (5′-3′)** | **Position (nt)** | **Length (bp)** |
| --- | --- | --- | --- | --- |
| JS27 | 5’ RACE Inner Primer | CGCGGATCCACAGCCTACTGATGATCAGTCGATG | 1-460 | 460 |
|  | 5’-460R | AGAAAGTGATCTCTCTATCCTCAGC |  |  |
|  | 330F | CTTCTGTGGCCTTTAAGCTGAATA | 330-3300 | 3000 |
|  | 3300R | CCAAACTGACATCCAAACCTATGT |  |  |
|  | 4300F | CTGTAGCGGTAGAAGTCTACGACAT | 4300-7300 | 3000 |
|  | 7300R | ATAGTCCTACCATCCGAACTGAATA |  |  |
|  | 6900F | AAGAATCCTGGACTGGCTCTATGTA | 6900-9800 | 2900 |
|  | 9800R | CAAGTGTCTATCTAGTGTCAAGGCA |  |  |
|  | 9100F | ATAGATGCCCTAAGGATGTATGTTT | 9100-12500 | 3400 |
|  | 12500R | CTAATACAACGGTTCCCCAGTAATC |  |  |
|  | 12000F | TGTATGATAGTCGTTACCGATACCC | 12000-14000 | 2000 |
|  | 14000R | CGAACTCATACCACTCACTATCTCAA |  |  |
|  | 13500F | AGAGACAGCGGTTTACAGGGTGATAT | 13500-16500 | 3000 |
|  | 16500R | GTGTGTGACATCTGATGAGGTTGAC |  |  |
|  | 15600F | GGTGGTACTACAATGGTTATGGTCC | 15600-18660 | 3000 |
|  | 18660 | GCTTCGACACAGAATTACAACACTC |  |  |
|  | 3’-18260F | ACWMACAYCTTCGAGAATCTCAAT | 18260-18899 |  |
|  | 3’ RACE Inner Primer | CGCGGATCCTCCACTAGTGATTTCACTATAGG |  | 740 |
| WH4-2 | 5’ RACE Inner Primer | CGCGGATCCACAGCCTACTGATGATCAGTCGATG | 1-450 | 450 |
|  | 5'-450R | GATCTCTCTATCCTCRGCGCCGAAGTAAA |  |  |
|  | 300F | GCTGAGGATAGAGAGATCACTTTCT | 290-870 | 560 |
|  | 870R | GCAAGAAAGCCTCATAATGGTAAC |  |  |
|  | 850F | ATCAAAGGTTACCATTATGAGGCT | 750-1700 | 850 |
|  | 1700R | AAAGAGTGGTTGAATGAAAGTGGT |  |  |
|  | 1500F | CTATTCGTAGGTTGCGTAGAAAGA | 1500-2400 | 900 |
|  | 2400R | ACAGGTTTACCAGTCACCTCAGAA |  |  |
|  | 2000F | ATGGATCTTAGTTGGGTTAAGCAC | 2000-4300 | 2300 |
|  | 4300R | TACCTGACACCRTCCTTMGGATC |  |  |
|  | 4000F | CMGAATTGAARCTYAAGTTYGTRCC | 4000-5800 | 1800 |
|  | 5860R | AGYCCTAARCACACACCAAA |  |  |
|  | 5586F | CTATTCGTAGGTTGCGTAGAAAGA | 5500-7200 | 1700 |
|  | 7300R | ACAGGTTTACCAGTCACCTCAGAA |  |  |
|  | 7050F | GGGTTMSTMMRGAYWTGTTYAAYGCTG | 7000-10500 | 3000 |
|  | 10500R | CCATCTCACAAGYTTTTATRTAAACRTCT |  |  |
|  | 9920F | TCAAGATAGGAAACCHACTSTTCAAG | 9920-12850 | 2900 |
|  | 12850R | CCGTAGTCAATACCTATGATCGTCATA |  |  |
|  | 12465F | ATGGATCTTAGTTGGGTTAAGCAC | 12460-13970 | 1500 |
|  | 13970R | TACCTGACACCRTCCTTMGGATC |  |  |
|  | 13600F | CCAATCTGTWACTGTATCAGRTACCAACG | 13600-15470 | 1800 |
|  | 15470R | AGYCCTAARCACACACCAAA |  |  |
|  | 15270F | CGATAGTCAATTGGCTATTGTCAGCGAAC | 15270-17600 | 2400 |
|  | 17655R | ACAGGTTTACCAGTCACCTCAGAA |  |  |
|  | 17400F | GCRTCKSAGATAAGTGTAACAGTAATCAC | 17400-18780 | 1300 |
|  | 18780R | TACCTGACACCRTCCTTMGGATC |  |  |
|  | 3'-18600F | CTATCTGAAYAGYCCGTTCAGAGAGCT | 18600-18858 | 300 |
|  | 3’RACE inner primer | CMGAATTGAARCTYAAGTTYGTRCC |  |  |
| WH4-1 | 2500F | GGACACTTTTCTTATAGTCTCGATAACGCC | 2500-4900 | 2400 |
|  | 4900R | AGCTCTAGGAACCGTCACGTGAACAATAG |  |  |
|  | 4700F | TCGACGGTCAACTATTCCATTCGGAGT | 4700-7500 | 2800 |
|  | 7550R | GTCTATGTCCTCGACCTTGTCTGCCATGA |  |  |
|  | 7210F | AGGTCTATATCAAAGTCGTGTAAGCCG | 7200-9500 | 2300 |
|  | 9520R | GCTGAGACGTGAATACAGCATCATCTTG |  |  |
|  | 9200F | CTAACTATTACGACGTTATGGCCGTAG | 9200-12200 | 3000 |
|  | 12200R | TAAGCGACGGCCAAGGATAAGTCTT |  |  |
|  | 12000F | GCTAAGCACAAACGCAGATCTAAGA | 12000-15300 | 3300 |
|  | 15300R | CACACATCTTGCTAATAAGACCTAAG |  |  |
|  | 15000F | CTGATTTCGACACGCGTGAATATTCTCGT | 15000-18000 | 3000 |
|  | 18000R | GTAGCACCGCTTAATAGAATAATTAATGTC |  |  |
|  | 18530F | CTATCTGAAYAGYCCGTTCAGAGAGCT | 18530-18860 | 300 |
|  | \| 3’RACE inner primer \| 试剂盒提供 \| \| --- \| --- \| | CMGAATTGAARCTYAAGTTYGTRCC |  |  |
| Z2 | 5'RACE inner Primer | CGCGGATCCACAGCCTACTGATGATCAGTCGATG | 1-500 | 500 |
|  | 500R | TCAAAGGCGAGTGCTCGAAGAATCTA |  |  |
|  | 4120F | TWCGDGCKGTRGAGARYACNCT | 4100-5200 | 1100 |
|  | 5270R | ATAGTCGAAGCAGAGTCGTCTATGGCA |  |  |
|  | 5150F | ATGACRTGGYTGAGAAAYAAYTCRGC | 5100-7400 | 2300 |
|  | 7480R | TCAGGTGTTAGACCTACATCTGGACA |  |  |
|  | 8950F | GACGARATWTTCYTRATGCATCTCGGTC | 8900-10900 | 2000 |
|  | 10950R | TCCCAATCRTTGTAHGGTTTMCCWAGCT |  |  |
|  | 10800F | CCYGACTTTAGTCATGARTTRATGGTSG | 10800-12400 | 1600 |
|  | 12420R | TCATACAGCTGATCAGCGATCAGAGAG |  |  |
|  | 14690F | GCRGAGTAYTTRATGTTRTGTGCKTAYG | 14600-16200 | 1600 |
|  | 16220R | TACATCAGWGCGATTAGAAGTGCAAGGA |  |  |
|  | 15470F | GATCACAGCCAACATACTATATCTGCCT | 15400-18700 | 3300 |
|  | 18780R | AAGCTCTCTGAACGGRCTRTTCAGATAGT |  |  |

D: G/A/T, W: A/T, R: A/G, H: A/T/C, Y: C/T, M: A/C, K: G/T, S: C/G

**TABLE S4** Origins, species and symptoms of kiwifruit samples and RT-PCR results of AcV-1 and other five known viruses

| Origin | *Actinidia* sp. | Sample ID | Symptom | AcV-1 | AcCRaV | CLBV | ASGV | AcVA | AcVB |
| --- | --- | --- | --- | --- | --- | --- | --- | --- | --- |
| Jiangxi | *A. chinensis* | JX5 | CL, Y | + | - | - | - | - | - |
|  |  | JX6 | / | + | - | - | - | + | - |
| Chongqing | *A. delicious* | C2 | CS | + | - | - | - | - | + |
|  |  | C4 | CS | + | - | + | - | - | - |
|  |  | C6 | CL | + | - | + | - | - | - |
|  |  | C8 | MF | + | - | - | - | + | + |
|  |  | CQ2 | CS | + | - | + | + | - | - |
|  |  | CQ6 | CRS | + | - | + | - | - | - |
|  |  | CQ20 | CS, MF | + | - | - | - | - | - |
|  |  | CQ25 | CL, Y | + | + | - | - | - | - |
|  |  | CQ26 | CL | + | + | - | - | - | + |
|  | *A. chinensis* | C7 | MF | + | - | - | - | - | + |
|  |  | CQ11 | CL | + | - | - | - | - | - |
|  |  | CQ17 | CL | + | + | - | - | - | + |
|  | *A. arguta* | CQ18 | / | + | + | - | + | - | + |
|  | Unknown | CQ19 | CL | + | - | - | - | + | + |
|  |  | CQ27 | CRS | + | + | - | + | - | - |
| Zhejiang | *A. chinensis* | GZ2 | CL | + | - | + | - | + | - |
|  |  | GZ6 | CL | + | - | + | - | - | - |
|  |  | GZ7 | CL, MF | + | - | + | - | - | - |
|  | *A. eriantha* | YD10 | CL, MF | + | - | - | - | - | - |
| Hubei | *A. chinensis* | JS10 | CRS, M | + | + | - | - | - | - |
|  |  | JS14 | CRS | + | + | - | - | - | + |
|  |  | JS15 | M | + | + | - | - | - | - |
|  |  | JS16 | MF | + | + | + | - | + | + |
|  |  | JS17 | CRS | + | - | - | + | - | - |
|  |  | JS22 | CL, M | + | + | - | - | - | - |
|  |  | JS23 | CL | + | + | + | + | - | + |
|  |  | JS24 | MF | + | - | - | - | - | - |
|  |  | JS25 | MF | + | - | - | - | - | - |
|  |  | JS27 | CL, MF | + | - | + | - | - | - |
|  |  | JS28 | CL | + | - | + | - | - | - |
|  |  | JS29 | CL, MF | + | - | + | - | - | + |
|  |  | JS31 | CRS | + | - | + | - | - | - |
|  |  | JS33 | MF | + | - | + | - | - | - |
|  |  | JS34 | CRS | + | - | + | + | - | + |
|  |  | JS35 | CRS | + | - | + | + | - | - |
|  |  | JS37 | / | + | - | - | + | + | - |
|  |  | JS40 | CRS | + | - | + | + | - | - |
|  |  | HB7 | CL, M | + | + | - | - | - | - |
|  |  | HB38 | MF | + | - | - | - | + | + |
|  |  | GCS14 | CRS | + | - | - | - | + | - |
|  |  | HB1 | / | + | + | - | - | - | - |
|  | *A. arguta* | WH4 | M, MF | + | + | - | - | - | - |
|  | Unknown | JS45 | CRS | + | - | + | - | - | - |
|  |  | JS47 | CL,M | + | - | + | - | - | - |
|  |  | SY2 | CL | + | + | - | - | - | - |
|  |  | SY4 | / | + | - | + | - | - | - |
|  |  | SY7 | CRS, MF | + | + | + | - | - | - |
|  |  | GCS3 | CRS | + | - | + | - | - | - |
|  |  | GCS6 | CRS, MF | + | + | - | - | - | - |
| Shandong | *A. chinensis* | YT11 | CRS | + | - | + | - | - | - |
|  |  | YT12 | CRS, MF | + | - | - | - | - | - |
|  |  | YT13 | CRS | + | + | - | - | - | - |
|  |  | YT14 | CRS | + | - | + | - | - | - |
|  |  | YT15 | CRS, MF | + | + | + | - | - | - |
|  |  | YT16 | CRS, MF | + | - | - | - | - | + |
|  |  | YT17 | CRS, MF | + | + | - | - | - | - |
|  |  | YT18 | CRS | + | - | + | - | - | - |
|  |  | YT19 | MF | + | + | - | - | - | - |
|  | *A. delicious* | YT20 | CRS | + | + | - | - | - | - |
|  | Unknown | YT1 | CL, M | + | - | - | - | - | - |
|  |  | YT2 | CRS, Y | + | - | - | - | - | - |
|  |  | YT3 | CRS | + | - | - | - | - | - |
|  |  | YT4 | CRS | + | - | - | - | - | - |
|  |  | YT5 | CL, Y | + | - | + | - | - | - |
|  |  | YT6 | CL | + | - | + | - | - | - |
| Fujian | *A. eriantha* | FJ6 | CL | + | + | - | - | - | - |
| Henan | *A.delicious* | ZZ4 | CL | + | - | - | - | - | - |
|  |  | Z2 | CRS, MF | + | - | + | + | + | + |
|  |  | Z2-15 | CRS, MF | + | - | + | + | + | + |
|  | *A.arguta* | ZZ13 | MF | + | + | - | - | - | - |
| Shanxi | *A. chinensis* | SX1 | CL | + | + | + | - | + | + |
|  |  | SX3 | CRS | + | + | - | - | - | + |
|  |  | SX4 | CS | + | - | + | - | + | + |

Only samples infected by AcV-1 viruses are listed.

CL, chlorosis; CS, chlorotic spot; CRS, chlorotic ringspot; M, mottle; MF, malformation; Y, yellowing; /, asymptomatic

+, positive; -, negative.
